# Project DECIDE, part II: decision-making places for people with dementia in Alzheimer’s disease: supporting advance decision-making by improving person-environment fit

**DOI:** 10.1186/s12910-023-00905-0

**Published:** 2023-04-28

**Authors:** Janina Florack, Christina Abele, Stefanie Baisch, Simon Forstmeier, Daniel Garmann, Martin Grond, Ingmar Hornke, Tarik Karakaya, Jonas Karneboge, Boris Knopf, Gregor Lindl, Tanja Müller, Frank Oswald, Nathalie Pfeiffer, David Prvulovic, Aoife Poth, Andreas Reif, Irene Schmidtmann, Anna Theile-Schürholz, Heiko Ullrich, Julia Haberstroh

**Affiliations:** 1 Faculty V: School of Life Sciences, Department of Psychology, Psychological Aging Research (PAR), University of Siegen, Adolf-Reichwein-Str. 2a, 57068 Siegen, Germany; 2 Klinikum Siegen, Siegen, Germany; 3 University Medical Center of the Johannes Gutenberg University Mainz, Mainz, Germany; 4 Würdezentrum, Frankfurt Am Main, Germany; 5 Present Address: University Hospital Frankfurt, Frankfurt Am Main, Germany; 6 Goethe University Frankfurt, Frankfurt Am Main, Germany

**Keywords:** Dementia, Alzheimer’s disease, Memory clinic, Supported decision making, Capacity to consent, Belonging, Meaning of home, Patient empowerment, Autonomy

## Abstract

**Background:**

The UN Convention on the Rights of Persons with Disabilities, and the reformed guardianship law in Germany, require that persons with a disability, including people with dementia in Alzheimer’s disease (PwAD), are supported in making self-determined decisions. This support is achieved through communication. While content-related communication is a deficit of PwAD, relational aspects of communication are a resource. Research in supported decision-making (SDM) has investigated the effectiveness of different content-related support strategies for PwAD but has only succeeded in improving understanding, which, although one criterion of capacity to consent, is not sufficient to ensure overall capacity to consent. The aim of the ‘spatial intervention study’ of the DECIDE project is to examine an innovative resource-oriented SDM approach that focuses on relational aspects. We hypothesise that talking to PwAD in their familiar home setting (as opposed to a clinical setting) will reduce the complexity of the decision-making process and enhance overall capacity to consent.

**Methods:**

People with a suspected or confirmed diagnosis of dementia in Alzheimer’s disease will be recruited from two memory clinics (*N* = 80). We will use a randomised crossover design to investigate the intervention effect of the decision-making place on capacity to consent. Besides reasoning capacity, which is part of overall capacity to consent and will be the primary outcome, various secondary outcomes (e.g., other aspects of capacity to consent, subjective task complexity, decisional conflict) and suspected moderating or mediating variables (e.g., meaning of home, demographic characteristics) will be assessed.

**Discussion:**

The results of the study will be used to develop a new SDM strategy that is based on relational resources for PwAD. If a change in location achieves the anticipated improvement in capacity to consent, future research should focus on implementing this SDM strategy in a cost-effective manner in clinical practice.

*Trial registration*: DRKS00030799.

**Supplementary Information:**

The online version contains supplementary material available at 10.1186/s12910-023-00905-0.

## Background

### Relevance

Article 12 of the UN Convention on the Rights of Persons with Disabilities (UN-CRPD, ratified by Germany in 2009) requires that people with disabilities should have access to the support they need to make legally valid decisions. The reform of the guardianship law in Germany (which entered into force on the January 1^st^, 2023) emphasizes the requirement to support the self-determination of people with disabilities.

The decline in the cognitive abilities of people with dementia in Alzheimer’s disease (PwAD) can result in them losing their capacity to consent and consequently their ability to make legal decisions that affect them [1, 2]. According to Grisso and Applebaum [3], the mental capacity to consent requires the ability to ‘understand’, ‘appreciate’, ‘reason’, and ‘express a choice’. PwAD lack the ability to ‘understand’ and ‘reason’ [4], while their ability to ‘appreciate’ and ‘express a choice’ are less impaired [2, 5, 6].

Previous research into supported decision-making (SDM) in PwAD has focused on the use of SDM strategies like keyword lists, elaborated plain language, and visualisation to compensate for deficits in verbal retrieval and improve a person’s understanding of information [7]. We consider SDM to be a communicative process and base our understanding of it on the combined communication model of Haberstroh et al. [8], in which the mentioned strategies support both verbal and non-verbal content aspects of communication. Poth et al. [9] have shown that such content-related SDM strategies, although leading to a significant enhancement in PwAD’s understanding of information, did not achieve a clinically relevant improvement in overall capacity to consent. The question facing us is therefore what else we can do to support decision-making processes in PwAD?

Besides verbal and non-verbal content aspects, the non-verbal relationship aspect is an important component of communication [8, 10]. Examples of these are feelings, mental states, and expressions of emotion [11]. Knebel et al. [10] showed that people with mild and moderate dementia also performed better in relationship aspects of communication than in content-related aspects.

From the viewpoint of environmental gerontology, decision-making is an interaction between characteristics of the person and the environment. Oswald and Wahl [12] postulated a framework that distinguishes between behaviour-driven ‘agency’ processes and experience-driven ‘belonging’ processes. As familiar places and objects provide a source of stability and self-continuity, particularly at a time of cognitive decline, Niedoba [13] highlights the importance of belonging for people in the transition to Alzheimer’s disease.

The current study will combine these findings and investigate an innovative SDM approach that focuses on the strengths of PwAD by supporting the relational aspects of communication and activating processes of belonging [14]. As part of the DECIDE project, the ‘spatial intervention study’ will investigate whether talking to PwAD in a highly familiar environment like their home enhances their capacity to consent. We expect the sense of belonging at home to reduce cognitive workload and the complexity of the decision-making process.

### Study aims

The *primary aim* of the spatial intervention study is to demonstrate that *home is a better place and more amenable for complex decision-making processes than an unfamiliar place* like a memory clinic, and that it improves the capacity to consent required (in this case) to create an advance directive. We assume that being at home, where the sense of belonging is strong, lessens subjective task complexity, decisional conflicts, and anxiety, and consequently increases rated capacity to consent. Our *secondary aim* is therefore to explore whether the anticipated influence of the place a decision is made on subjectively perceived task complexity, decisional conflicts and anxiety actually exists and, if so, whether it is affected by the extent of a person’s sense of belonging at home. We will also explore the influence of further potential confounders, moderators, and mediators.

## Methods

### Participants

Participants will be recruited from two outpatient memory clinics (University Hospital Frankfurt, Germany; Klinikum Siegen, Germany). We plan to recruit *n* = 40 participants at each location, for a total of *N* = 80 participants (for calculation see below). Recruitment is terminated either after reaching the optimal sample size or by the end of the project duration (planned for March 31st, 2024).

We will include patients with a suspected or confirmed diagnosis of dementia in Alzheimer’s disease (F00.1), or a mixed type of dementia in Alzheimer’s disease (F00.2). The neuropsychological diagnosis will be the determining factor in differentiating between Alzheimer’s disease and other types of dementia. The responsible neuropsychologists will analyse the cognitive profiles of potential participants and discuss complex cases with experts from their research group where appropriate. Since capacity to consent to study participation may be an issue, the inclusion process will be based on the decision tree for the inclusion of *non-consenting* individuals in medical research [15]. This will permit us to include participants whose capacity to consent is questionable.

Exclusion criteria are severe dementia (confirmed clinical diagnosis or MMSE-score < 10), delirium, intellectual disability, severe mental illness (e.g., clinical diagnosis of severe depression or GDS-score > 10), lack of capacity to consent to medical research with simultaneous inability to participate in SDM involving a relative or proxy, no assent by the patient, uncompensated and pronounced sensory deficits, or persons whose knowledge of the German language is insufficient to understand the study documents and/or an interview. They will also be excluded, if they have taken part in the intervention phase of the first study of the DECIDE project [16]. There are no restrictions regarding concomitant interventions.

### Intervention

The intervention lies in making decisions at home, which is a place where patients have a strong sense of belonging, as opposed to a memory clinic where they do not. It is important to note that our focus is on capacity to consent. Its object, the creation of an advance directive, is only an example of a relevant and complex decision-making situation. The offer to participate in advance care planning (ACP) and the ACP itself are *not* part of the intervention.

### Study design

We will use a randomised AB/BA crossover design to study the effect of the spatial intervention ‘decision-making place: home vs. memory clinic’ on capacity to consent to an advance directive. Each participant will undergo one session at the memory clinic and one session at their home. The decision where the first session will be conducted will be made randomly for each PwAD, and stratified by site. The crossover design will further permit us to examine the presence of substantial period and carryover effects.

### Procedure

#### Data collection

**Fig. 1:**
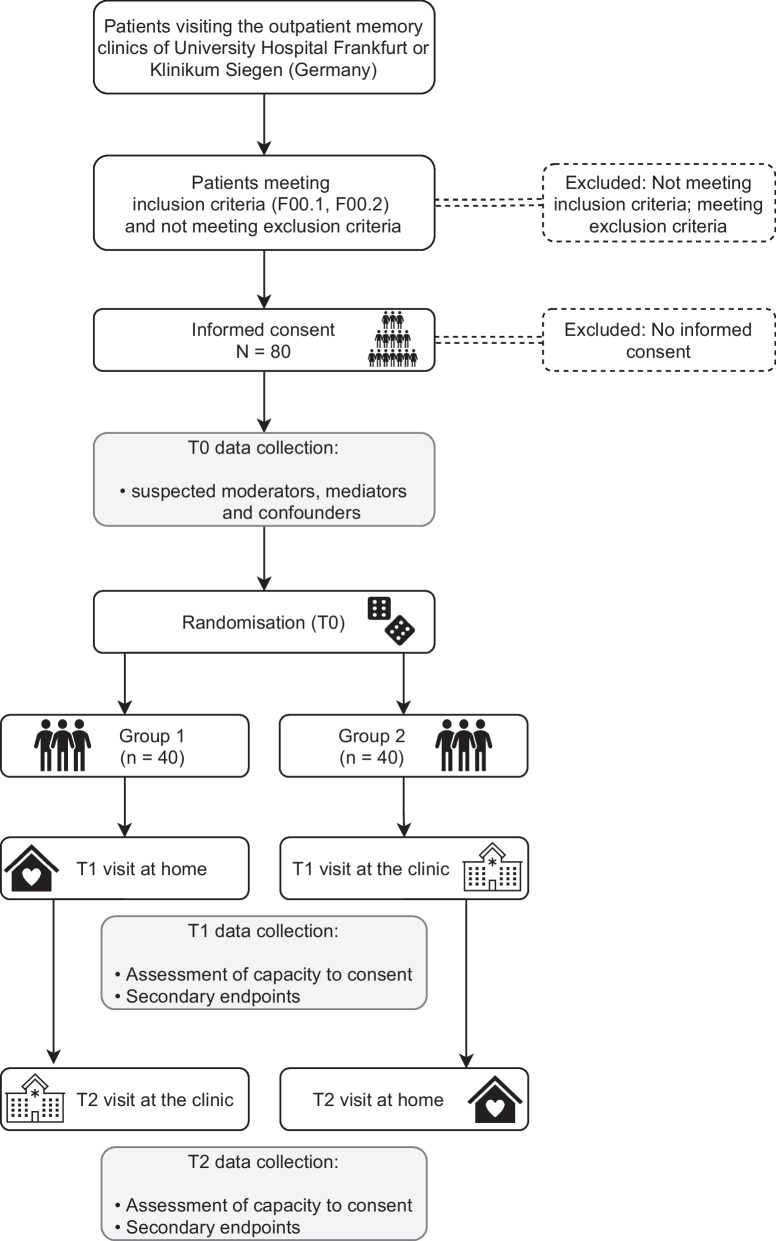
Overview of the study design and course of the spatial intervention study. T0 is the final appointment of the standard diagnostic investigation at the memory clinic. T1 & T2 are additional appointments for research interviews

The procedure of the spatial intervention study is presented in Fig. 1*.* Patients fulfilling the inclusion criteria will be asked whether they wish to participate at their final appointment at the memory clinic (T0). If they agree, the physician will assess suspected moderators, mediators and confounders including demographic variables, severity of dementia and depression, perceived sense of belonging to home, need for autonomy in medical decisions and health literacy (see Table 1). Afterwards, two further appointments will be made. Patients will be assigned to one of the two groups by using site specific randomisation lists with two levels (i.e., the order of visits) and a final block size of 4. These lists were created using R and the R package blockrand [17, 18]. At the beginning of each session (T1 & T2), the research physician will assess the capacity to consent of the individual to complete an advance directive, as well as subjective task complexity, decisional conflicts, and anxiety in the decision-making situation (see Table 1). As the decision-making situation should be as realistic as possible and also be of benefit to the participants, they will all have the chance to take part in ACP following the assessment. They can decide whether to create an advance directive with the help of the study physician, or whether to gather information on a possible care directive or lasting power of attorney. It is also possible to decide against ACP. The ACP process will be divided between the two sessions to avoid overload.

**Table 1:** Endpoints, potential predictors and measuring instruments

Measured quantity	Instrument and description	Time of assessment
Ability to ‘understand’, ‘appreciate’, ‘reason’ and ‘express a choice’ as preconditions of capacity to consent	CAT-AD: adjusted version of the DCAT-PAD/MacCAT-T [20, 21] for PwAD	T1 & T2
	Semi-structured interview	
Subjective task complexity	Subscale ‘post task: subjective task complexity’ [28]	T1 & T2
	4-item-subscale	
Decisional conflicts	Subscale ‘uncertainty’ of the German version of the ‘Decisional Conflict Scale’ [29]	T1 & T2
	3-item-subscale	
Anxiety in the decision-making situation	Single item described by LeBlanc et al. [30]	T1 & T2
Age, gender		T0
Education	Single item based on German education system	T0
Severity of dementia	Mini-Mental Status Examination [31]	T0
	Screening for cognitive abilities to detect cognitive impairment or dementia	
Severity of possible depression	Geriatric Depression Scale [32]	T0
	Screening for depression in the elderly	
Perceived sense of belonging to home	Selection of items from Meaning of Home Questionnaire [26, 27]	T0
	Adjusted for people with dementia & validated during the project	
	Guided interview	
Need of autonomy for medical decisions	Single item described by Strull et al. [33]	T0
Health literacy	Single item described by Morris et al. [34]	T0

As in our previous studies, the presentation of information will be standardised for both the intervention and the control settings in order to ensure as much consistency as possible in everything apart from the ‘place’. Consequently, all support strategies that have proven to be effective in our own and international studies (e.g., elaborated plain language, key word list, time of day: 8–15 o’clock, person-centred attitude), will be used in both places (as recommended in the current AWMF-S2k-guideline “Einwilligung von Menschen mit Demenz in medizinische Maßnahmen”; for a summary of available evidence on SDM for PwAD, see [7, 19]).

#### Adherence, retention, and withdrawal

In order to avoid selective drop-out due to the cognitive impairments of the participants, we have different strategies to ensure that they meet their appointments. When they agree to participate in the study, they receive a reminder note with the dates and location. Furthermore, we will call the participants and, if necessary, their relatives two days in advance to ensure that they do not forget their appointment. In case of a missed appointment, we will offer to make up for it on a later date. If a person rejects participating in the study or decides to end it early, we try to record the reasons for their rejection or cancellation.

### Outcomes

#### Primary endpoint

The primary endpoint in this study will be an assessment of capacity to consent to create an advance directive both at home and at the clinic using the Competence Assessment Tool—Advance Directive (CAT-AD). To create the CAT-AD, we adapted both the MacCAT-T [20] and the DCAT-PAD for advance directives in PwAD [21]. The MacCAT-T is a well-known international instrument for the standardised assessment of the capacity to consent to treatment options [22] and the DCAT-PAD is a version of the MacCAT-T that has been adapted for psychiatric advance directives. Both tools test participants’ ability to ‘understand’, ‘appreciate’, ‘reason’ and ‘express a choice’. As we expect the intervention to particularly support patients’ reasoning capacities, the corresponding subscore will be the primary endpoint of the study. The other subscores and the total score will be secondary endpoints. In view of our target population, we also reduced the complexity of the scale ‘*Understanding Risks and Benefits of Advance Directives’*. To meet German legal standards for informed consent, we added a further item to the scales *‘Appreciation’* and *‘Expressing a Choice’*. To compensate for the dominance of verbal recall shown in previous studies [23, 24], the information is presented to the participants in the form of bullet points [25].

#### Secondary endpoints and suspected moderators, mediators, and confounders

Besides the CAT-AD scores mentioned above, we will assess several secondary endpoints, as well as suspected moderators, mediators and confounders. Table 1 provides an overview of all endpoints and covariates, along with the corresponding measuring instruments. Further secondary outcomes are subjective task complexity, decisional conflicts and anxiety in the decision-making situation. Suspected moderators, mediators and confounders are severity of dementia and – if present – depression, the perceived sense of belonging to home, the need for autonomy in medical decision-making, health literacy, and such sociodemographic variables as age, gender, and education. The listed instruments were adjusted for the present study and our sample as appropriate. To assess the perceived sense of belonging at home, the ‘Meaning of Home Questionnaire’ [26, 27] will be revised and validated for PwAD as part of the DECIDE project. For the spatial intervention study, we will select the items that are most suitable for measuring the extent of perceived belonging based on theoretical and statistical considerations.

### Statistical methods

#### Analysis population

Since our intervention consists in making a decision at two different places in a certain order, the only possible reason why the intervention might not conform to the study protocol is – apart from missing data issues – that the sequence of the places is swapped. If this occurs, it will be due to an organisational error, i. e. completely at random. This means the patient can be analysed with the sequence group that corresponds to his or her actual sequence without generating any bias. Apart from this, as the appointments for the interviews involve two persons (study nurse and physician) checking the correct sequence independently, we consider such an error highly unlikely. Hence, the analysis population will be the “as treated” population.

#### Statistical analysis

A linear mixed model with place, period, and interaction of place and period as fixed effects and patient as a random effect will be used to analyse the primary endpoint. We will check for carryover by testing the interaction between place and period. If there is no significant interaction effect, differential carryover can be neglected. Otherwise, statistical analysis will be stratified by period, whereby the main analysis will be restricted to the first period, and data from the second period will be used for sensitivity analysis.

An exploratory investigation of the impact of the place the decision is made on subjective task complexity, decisional conflicts and anxiety will be based on a model that is analogous to the main analysis.

Furthermore, both linear mixed models will be extended to include an exploratory analysis of potentially confounding, moderating, and mediating variables.

The relationship between the place the decision is made, and the other secondary endpoints will be explored by conducting two-sample comparisons between the sequence groups in a two-step procedure based on Wilcoxon-Mann–Whitney tests, consisting of a pretest for differential carryover and a main test for the location effect. This is essentially equivalent to the mixed model approach but assumes no particular probability distributions and does not permit inclusion of covariates. If the pretest is significant, the main test will only be performed on data from the first period.

The features of the patient sample will be summarised descriptively. Absolute and relative frequencies will be used for categorical variables, and mean, standard deviation, minimum, maximum, and quartiles for metric variables.

#### Missing data

From our experience with the first part of the DECIDE project, we expect a low overall proportion of missing data in the baseline assessment and the first ACP interview, which we will handle using complete case analysis (with less than 5% of incomplete records) or multiple imputation. A higher proportion might accrue in the second interview. As this kind of missingness would still be at random (conditional of the observed period variable), it can be resolved using multiple imputation, too. Apart from this, should it turn out that too many patients are available for the first ACP interview only, we will restrict our analysis to these observations as described in the next section.

#### Calculation of sample size

From a recent trial evaluating SDM strategies for patients with dementia (EmMa project) [9], the MacCAT-T subscale scores of the 30 patients with Alzheimer’s disease (14 in the intervention, 16 in the control group) were used to define the relevant standardised location effect for the sample size calculation. While the SDM strategies in EmMa project only increased scores on the “understanding” subscale of the MacCAT-T, we expect that in our trial, the support of the familiar environment of the patient’s home will rather influence the “reasoning” subscale. Thus, we anticipate that as a result of the DECIDE intervention, the change in the reasoning score will be similar to the standardised effect of 0.582 found for understanding in EmMa project.

Due to memory limitations in the study population, we do not expect any substantial carryover effect. The relevant quantity for the sample size calculation will therefore be the location contrast estimated from both periods. However, although unlikely to be detected in a cross-over design powered for the main effect of the place the decision is made, slight differential carryover will still bias the effect. Hence, the sample size should allow us to compensate for power loss due to undetected differential carryover of up to 20% of the spatial intervention effect.

Use of a cross-over design further means that only intra-individual variance must be taken into account. The stronger the correlation between a patient’s two observations, the smaller the intra-individual variance and the smaller the necessary sample size. Although we expect some positive correlation, we will assume uncorrelated observations to be on the safe side.

Based on these assumptions, and with a two-sided significance level of α = 5%, the power.t.test () function of the R stats package (version 4.0.2) results in a total sample size of 80 patients, 160 observations, for a power of 90%. If recruitment difficulties arise, 80% power will still be achieved with 60 patients and 120 observations. If – as is to be expected – a positive correlation exists between the same patient’s measurements, even fewer patients will suffice to reach 80% power. For example, assuming a within-patient correlation of 0.2, the inclusion of 48 patients and 96 measurements will mean the power is still 80%. If a significant proportion of patients can only be studied once, the analysis could be restricted to the first observations. However, if 80 first measurements are available, the power for detecting the location effect will be reduced to 58%. In this case, we would try to include a further 16 patients and thus achieve 80% power.

**Fig. 2:**
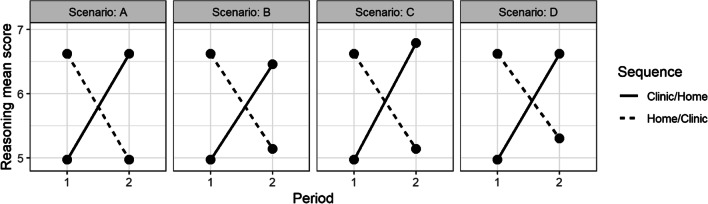
Scenarios considered in the simulation. Scenario A is the basic scenario with only a location, but no carryover or period effects. Scenarios B and C include a carryover and a period effect amounting to 20% of the location effect respectively, and in scenario D, both are included. The standard deviation of the score is 2.83 in all scenarios

Since a two-step evaluation procedure based on a linear mixed model will be used to analyse the primary endpoint, we verified the sample size in a simulation of the procedure with 10,000 analysis runs per scenario. Based on the relevant standardised effect (see above) and the variance in the ‘reasoning’ scores observed in EmMa project [9], we considered four scenarios covering a range of realistic outcomes. They are presented in Fig. 2. The simulation confirms the sample sizes obtained from the t-test formula.

### Quality control

#### Data management

The Data Management Committee (DMC) consists of a biostatistician and a data manager, neither of whom are involved in data collection. They are responsible for data digitalisation, data quality assurance and privacy protection, and for providing statistical advice and surveying the database. Further, they created the randomization list for allocating the participants to the different intervention groups. The paper-based recorded data will be digitalised in the LimeSurvey online survey tool. To monitor the data, a double data entry will be performed on one randomly selected record out of each set of ten successive records.

#### Trial monitoring

A Trial Management Committee (TMC) will be responsible for elaborating the study design and procedure, data collection and informed consent materials where appropriate. It will meet twice a week to discuss and solve current problems and initiate any necessary steps. The TMC will include the Steering Committee (SC), which organises and moderates TMC meetings, reviews the study’s progress and agrees on changes to the study protocol that become necessary during the course of the study. The SC will keep the study protocol updated and communicate all changes to the study members and the ethics committee.

#### Principal and lead investigators

The principal investigator designed the study and acquired the grant. Being the head of the SC, she is responsible for all final decisions during study implementation. She will be responsible for most of the communication with the associated partners. She declares to have no competing interests.

Lead investigators are the study physicians and neuropsychologists at each of the study centres. As part of the TMC, they represent the link between research and practice and are responsible for data collection.

#### Audits

Members of the Institute for Medical Ethics and History of Medicine at the Ruhr-University Bochum, Germany, form an independent ethics advisory board for the study. They will advise us on questions related to research ethics.

We plan to organise biannual expert workshops to obtain scientific advice from international experts from different disciplines. The topics will relate to challenging issues during the research process, e.g., clinical diagnostics of dementia, decision-making, task complexity, capacity to consent. Additionally, our cooperation partner, the Frankfurt Forum of Interdisciplinary Ageing Research, will provide theoretical input on relevant gerontological issues such as person-environment processes in aging.

Finally, we have established a patient advisory board in cooperation with the Alzheimer Society Siegen at our department. Its function is to advise researchers on practical and ethical questions that arise during the research process. We can thus adapt our research to the needs of PwAD. The patient advisory board currently consists of four patients and meets monthly. In the DECIDE project’s study presented here, we will ask the advisory board for advice on ethical questions and questions related to the study procedure or materials.

### Ethics and dissemination

The study procedure and materials have been reviewed and approved by the Ethics Committee of the Medical Council Westfalen-Lippe, the medical faculty of University of Münster (trial no. 2022–080-f-S) and the Ethics Committee of the Medical Faculty at Goethe University Frankfurt am Main (trial no. 2022–853). The study has been registered with the Germany Registry for Clinical Trials (DRKS, Deutsches Register Klinischer Studien, no. DRKS00030799). All items of the WHO Trial Registration Data Set can be found in Additional file 1. Any relevant amendments to this protocol will be communicated formally to the study registry, and to the ethics committees for approval.

#### Possible harm, ancillary, and post-trial care

In addition to routine clinical procedures at the memory clinic, participants will have two study appointments. At the beginning of each appointment, their capacity to consent to an advance directive will be assessed using standardised methods during a meeting lasting around 20 min. As the complexity of this decision-making situation is expected to be stressful for the PwAD, we will use the support strategies to minimise it. Afterwards, the PwAD can decide whether to create an advance care plan or not. The creation process will be spread over two appointments to avoid overload. Since the ACP is not part of the study, it can be tailored to the individual needs of each participant.

#### Informed consent and assent

At their final appointment at the memory clinic, the study physician will tell the participants about the study and answer any questions they have. If participants agree to participate, he will obtain their written informed consent. The information provided to participants and the informed consent form are available on request.

Since participants have a suspected or final diagnosis of dementia in Alzheimer’s disease, their capacity to consent to study participation may be an issue. Following German AWMF S2k guidelines to medical procedures, we will support the participants as far as possible. If their capacity to consent is questionable, consent by proxy will be an option but nonetheless require the patient’s assent. Patients that neither consent nor agree to consent by proxy will be excluded from study participation.

#### Confidentiality and access to data

At each of the three sessions (see Fig. 1), study documents will be filled out on paper without personal identifying information of participants. After each visit, paper documents will be scanned into the digital patient file and the originals will be destroyed. This procedure allows us to merge the three sets of anonymized documents. After completion, the relevant documents will be printed as anonymized records and safely stored in the respective memory clinic. After successful data entry and quality control, the printouts will be destroyed.

The DMC has access to all digitalised study data. Researchers graduating based on work on the DECIDE project will only have access to the data they require for their theses. The (anonymous) data used in publications will be accessible to other researchers via PsychData, a German data-sharing platform for psychological research (www.psychdata.de). Study protocols and codebooks will also be shared. After publication, the data will be made available to other researchers for six months to ten years if they have specific and reasonable requests for data usage and sign the data usage agreement.

#### Dissemination of results

It is our intention to publish findings from the study in open-access journals that enforce strict quality assurance processes (i.e., peer-reviewed journals). Every listed author will have made a substantial contribution to the manuscript. We do not intend to use any professional writers. We also plan to include our findings in the updated version of the AWMF-Guideline “Einwilligung von Menschen mit Demenz in medizinische Maßnahmen” (consent to medical treatment by people with dementia), and we will present the study results at relevant national and international conferences concerning dementia and geriatrics.

## Discussion

The current study will investigate an innovative and resource-oriented approach to SDM in PwAD. Past studies have only succeeded in improving the understanding of information by compensating for people’s impairments in verbal retrieval and have been unable to achieve any clinically relevant improvement in overall capacity to consent. We will focus on the resources of PwAD in the relational domain of communication by choosing their highly familiar home as a location for the decision-making process. We hypothesise that improving the fit between person and environment will reduce complexity and thus enhance capacity to consent. In contrast to past studies, we expect the new SDM strategy to facilitate the reasoning processes that are most challenging for PwAD. If the choice of the home as a location for decision-making has the desired effect, we expect use of the support strategy to be extended to include other important and complex decisions affecting PwAD, such as decisions on medical treatments, a final will, or on whether to move to a nursing home.

## Limitations

It will be challenging to recruit a sample of PwAD of sufficient size. However, as our study physicians provide regular care in a memory clinic, we have good access to the target population and to potential participants. Nevertheless, PwAD have limited capacities and would be required to attend two further appointments in addition to the three regular appointments at the memory clinic. As has frequently been the case in the past, this may result in our recruiting less impaired people because they have more resources and thus agree more often to study participation.

Moreover, it will not be possible to blind the study physicians to the intervention effect because they are part of the research team, and a change in location is an obvious intervention. We therefore risk bias in the assessment of capacity to consent if the study physicians unconsciously give participants higher scores either at home or in the clinic. To minimise the risk of bias, our physicians will be made aware of the possibility and use a standardised instrument for the assessment of capacity to consent.

Furthermore, the standardised assessment of capacity to consent to an advance directive using the CAT-AD is known to be more conservative than a clinical assessment [35]. Grisso and Applebaum [36] conclude that the assessment of the capacity to consent should not be wholly based on the instrument, and a sum score for overall capacity to consent should not be calculated. In our study, the final decision on whether a person is able to consent will therefore be made by using cut-off criteria at a subscale level.

### Outlook

Should the intervention prove successful, one central question for future studies will be how to implement the spatial intervention in clinical practice. In daily life, physicians working in memory clinics do not have the resources to make additional appointments with their patients at home. The economic use of the SDM strategy will therefore require persons that visit PwAD at home on a regular basis to play an important role. It is conceivable that general practitioners conducting home visits, caregivers, or dementia care nurses should play a role in the process. Future research could investigate the integration of our SDM strategy into overall dementia care management (DCM). DCM is a collaborative model of care in which qualified professionals support PwAD and their caregivers in a home setting according to their individual needs [37]. These professionals could be trained in the assessment of capacity to consent and in the use of SDM to support PwAD in complex decision-making situations.

## Supplementary Information

**Additional file 1**. WHO Trial Data Registration Set.

## Data Availability

Data and materials on which published results are based will be available from the corresponding author via the data sharing platform PsychData (www.psychdata.de) upon reasonable request.

## Abbreviations

PwAD: People with dementia in Alzheimer’s disease
SDM: Supported decision making
ACP: Advance care planning
DCM: Dementia Care Management
