# Supplementary material for: Project DECIDE, part II: decision-making places for people with dementia in Alzheimer’s disease: supporting advance decision-making by improving person-environment fit
Source: BMC Med Ethics. 2023 Apr 28;24:26. doi: 10.1186/s12910-023-00905-0 (PMC10148477; doi:10.1186/s12910-023-00905-0)
Supplement: Supplementary file 1 — Additional file 1. WHO Trial Data Registration Set. [file 12910_2023_905_MOESM1_ESM.docx]

**Appendix**

**WHO Trial Data Registration Set**

| **Category** | **Information** |
| --- | --- |
| Primary registry and trial identifying number | drks.de DRKS00030799 |
| Date of registration in primary registry | February 7^th^, 2023 |
| Source(s) of monetary or material support | German Federal Ministry of Education and Research |
| Primary sponsor | Universität Siegen |
| Secondary sponsor(s) | - |
| Contact for public queries | Janina Florack, Universität Siegen, 0049 (0)271 740-5008, janina.florack@uni-siegen.de |
| Contact for scientific queries | Julia Haberstroh, Universität Siegen, 0049 (0)271 740-4053, julia.haberstroh@uni-siegen.de |
| Title | Decision-making places for people with dementia in Alzheimer’s disease – part 2: Supporting advance decision-making by improving person-environment fit |
| Countries of recruitment | Germany |
| Health condition(s) or problem(s) studied | Lack of effective supported decision-making strategies for people with dementia in Alzheimer’s disease |
| Intervention(s) | Intervention: decision-making place home  No intervention: decision-making place memory clinic |
| Key inclusion and exclusion criteria | Inclusion criteria: - a suspected or confirmed diagnoses of Alzheimer’s dementia (F00.1) or mixed type of Alzheimer’s dementia (F00.2)  Exclusion criteria: - severe dementia - delirium - intellectual disability - severe mental illness - lack of capacity to consent to medical research with simultaneous inability to participate in supported decision making involving a relative or proxy - uncompensated and pronounced sensory deficits - knowledge of the German language is insufficient to understand the study documents and/or an interview |
| Study type | Interventional allocation: randomised controlled study Intervention model: crossover Masking: none Primary purpose: enhancement of capacity to consent |
| Date of first enrolment | March 2023 |
| Target sample size | 80 |
| Recruitment status | Recruiting |
| Primary outcome(s) | capacity to consent to an advance directive |
| Key secondary outcomes | Subjective task complexity Decisional conflicts Anxiety in the decision-making situation |
